# Has Metal-On-Metal Resurfacing Been a Cost-Effective Intervention for Health Care Providers?—A Registry Based Study

**DOI:** 10.1371/journal.pone.0165021

**Published:** 2016-11-01

**Authors:** Ruth Pulikottil-Jacob, Martin Connock, Ngianga-Bakwin Kandala, Hema Mistry, Amy Grove, Karoline Freeman, Matthew Costa, Paul Sutcliffe, Aileen Clarke

**Affiliations:** 1 Warwick Evidence, Division of Health Sciences, Warwick Medical School, University of Warwick, Coventry, United Kingdom; 2 Northumbria University, Department of Mathematics, Physics and Electrical Engineering, Faculty of Engineering and Environment, Newcastle upon Tyne, United Kingdom; Harvard Medical School/BIDMC, UNITED STATES

## Abstract

**Background:**

Total hip replacement for end stage arthritis of the hip is currently the most common elective surgical procedure. In 2007 about 7.5% of UK implants were metal-on-metal joint resurfacing (MoM RS) procedures. Due to poor revision performance and concerns about metal debris, the use of RS had declined by 2012 to about a 1% share of UK hip procedures. This study estimated the lifetime cost-effectiveness of metal-on-metal resurfacing (RS) procedures versus commonly employed total hip replacement (THR) methods.

**Methodology/Principal Findings:**

We performed a cost-utility analysis using a well-established multi-state semi-Markov model from an NHS and personal and social services perspective. We used individual patient data (IPD) from the National Joint Registry (NJR) for England and Wales on RS and THR surgery for osteoarthritis recorded from April 2003 to December 2012. We used flexible parametric modelling of NJR RS data to guide identification of patient subgroups and RS devices which delivered revision rates within the NICE 5% revision rate benchmark at 10 years. RS procedures overall have an estimated revision rate of 13% at 10 years, compared to <4% for most THR devices. New NICE guidance now recommends a revision rate benchmark of <5% at 10 years. 60% of RS implants in men and 2% in women were predicted to be within the revision benchmark. RS devices satisfying the 5% benchmark were unlikely to be cost-effective compared to THR at a standard UK willingness to pay of £20,000 per quality-adjusted life-year. However, the probability of cost effectiveness was sensitive to small changes in the costs of devices or in quality of life or revision rate estimates.

**Conclusion/Significance:**

Our results imply that in most cases RS has not been a cost-effective resource and should probably not be adopted by decision makers concerned with the cost effectiveness of hip replacement, or by patients concerned about the likelihood of revision, regardless of patient age or gender.

## Introduction

Hip replacement is a common elective surgical procedure. If an estimated 80,000 replacements are conducted annually at an average cost of £8,500 per procedure, this would cost the UK NHS approximately £680 million per annum [1, 2]. The overall post-operative costs are approximately five times greater than this due to hospital stay and post-operative support [3]. Approximately 80,000 procedures are performed each year and, assuming lifetime revision rates are about 5% and the average cost of revision surgery is approximately £17,000, prosthesis failure adds a further £68 million over subsequent years [4]. Total hip replacement is currently the most commonly used surgical intervention for end stage arthritis of the hip. In 2007 about 7.5% of UK implants were metal-on-metal joint resurfacing (MoM RS) procedures [5], but due to high revision rates and concerns about metal debris, the use of RS declined to about 1% of UK hip procedures in 2012. The DePuy Articular Surface Replacement (ASR) device (representing about 9% of UK RS) was recently withdrawn after unacceptably high revision rates were observed, and in 2015 Smith and Nephew voluntarily withdrew some Birmingham Hip devices (the most widely used RS device in the UK) on the grounds that in certain populations (e.g. women) devices with a relatively small head size failed to reach the NICE benchmark for revision. Similar concerns have led to the near abandonment of metal-on-metal THRs [6–9]. However some people still advocate for RS in the young and active, whose primary implant is expected to need replacement over their lifetime [1,10]. In particular, it has been argued that there may be a role for the best RS devices in highly selected patients such as active young men with a large femoral head size [1,11].

Updated NICE guidance for hip replacement recommends a 10 year revision rate benchmark of <5% [12]. The 11th National Joint Registry (NJR) report (2014) estimated that in the past the RS 10 year revision rate has been about 13% [5]. This compares unfavourably with 10-year revision rates for frequently used THR devices, which are less than 4% and indicates that, as a class, RS is unlikely to have been cost-effective relative to THR [12–14]. RS devices produced by at least sixteen different manufacturers have been used in the UK and they differ with respect to revision rate [5]. It remains an open question whether implantation of any RS devices or the best RS devices in highly selected patients such as active young men with a large femoral head size can be cost-effective.

We use data from the National Joint Registry for England and Wales to identify RS device-patient combinations likely to satisfy the NICE benchmark, and to address the question of whether past use of any RS devices, even those within the 5% revision rate at 10 years, has represented a cost-effective use of NHS resources.

## Methods

### Prosthesis categories

The National Joint Registry (NJR) for England and Wales supplied individual patient data (IPD) for RS and THR surgery for osteoarthritis, recorded from April 2003 to December 2012. Following the approach of others [5,12,13], we used flexible parametric modelling (Lambert and Royston 2009) [15] of NJR RS data to guide identification of patient subgroups and RS devices which delivered revision rates within the NICE 5% revision rate benchmark at 10 years. Variables investigated included the following: device manufacturer, RS head size, age at primary intervention, American Society of Anaesthetologists (ASA) grade, and sex Patients were coded in the NJR as: ASA grade 1 (“*normal healthy individuals*”); ASA 2 (“*mild systemic disease that does not limit activity*”); or ASA grades 3 to 5 (“*severe systemic disease that limits activity but is not incapacitating*”, “*incapacitating systemic disease which is constantly life threatening*”, and “*moribund*, *not expected to survive 24 hours with or without surgery*”). Patients who had a primary indication for surgery other than osteoarthritis were excluded along with those graded ASA 3, ASA4 and ASA5 (who represented only 3% of RS recipients), and those receving the DePuy RS device and data were then stratified according to sex…Devices produced by six of the sixteen manufaturers which contributed 96% of relevant implants were included, comprising Birmingham Hip, Biomet, Centerpulse, Corin, Finsbury and Wright UK. Where modelling indicated that patients and devices had satisfied the NICE benchmark, we pooled device and patient subgroups by manufacturer with the aim of estimating cost-effectiveness relative to THRs that satisfy the NICE benchmark.

Multiple manufacturers providing about 150 different THR components are listed in the NJR. The most commonly used THR devices (covering 62% of all THRs) were selected for potential comparison with RS on the basis of frequency of use of components (cup component group; cup component type; cup composition; cup fixation; cup implant type; head component type; head composition; liner component type; liner composition; stem component type; stem fixation; stem implant type) [12,16]. S1 Table summarises the characteristics of five frequently used THR devices which differ according to bearing surfaces, and/or mode of fixation. We have previously reported that all these devices have shown revision rates within the NICE benchmark [12].

### Economic model

**Fig 1:**
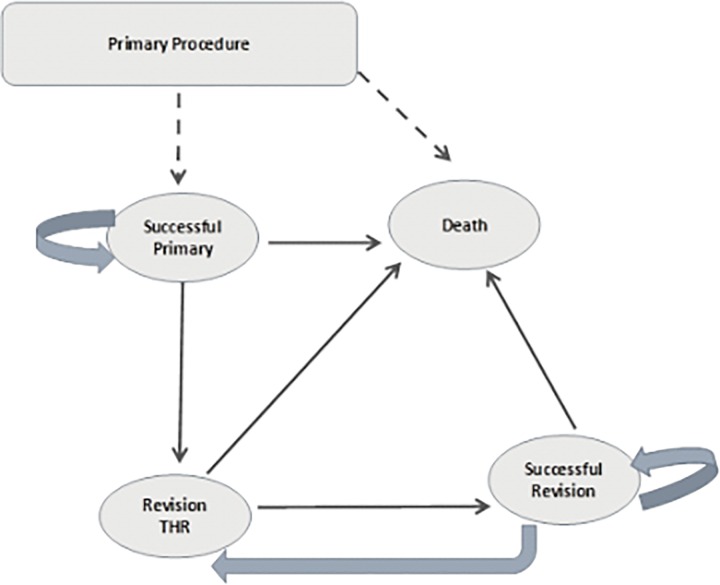
Semi-Markov model structure.

We used the well-established simple multi-state semi-Markov model developed by Fitzpatrick *et al*. (Fig 1) [17]; this model has been used repeatedly by others [14,18,19]. In the model, patients occupy one of four mutually exclusive health states: (1) successful primary THR or RS surgery (2) revision THR surgery (patients can move into this state more than once but stay in this health state for one annual cycle only); (3) successful revision THR surgery; (4) dead (patients may enter this state both due to operative mortality or due to death from other causes). We assumed that if the initial RS or THR required revision, the patient would be revised with a THR prosthesis.

Model inputs included the following: annual transition probabilities for time to revision and death, costs, and utilities of health states. The model cycle length was one year. We explored multiple parametric distributions to model lifetime revision rates using individual patient data from the NJR. The transition probability to death after cycle one was based on data from the Office for National Statistics for appropriately aged men and women [20]. An NHS and Social Services perspective was chosen for costs and a 3.5% annual discount rate was applied to both costs and health outcomes [21] All costs are reported in 2014 British Pounds. Table 1 summarises the model input parameters and sources [4,22–26].

**Table 1:** Model inputs for the base case analysis of cost-effectiveness.

Transition probabilities					
Health state	Mean value	SE			Source
Surgical mortality*	0.0050	0.001			NJR annual report^22^
Risk of re-revision	0.0518	N/A			Pennington *et al*^30^
**Utility inputs**					
**Utilities—Male**	**Mean value**	**SE**	**Beta distribution Parameter α**	**Beta distribution Parameter β**	**Source**
Age 40–50	0.736	0.0179	443	159	PROMS^24^
Age 50–60	0.767	0.0066	3133	952	
Age 60–70	0.762	0.0038	9112	2393	
Revision surgery	0.575	0.009	1496	1106	
**Utilities—Female**					
Age 40–50	0.720	0.0129	872	339	PROMS^24^
Age 50–60	0.742	0.0058	4287	1491	
Age 60–70	0.769	0.0032	13128	3944	
Revision surgery	0.553	0.007	2201	1779	
**Cost inputs**					
**RS versus THR**					
**Cost**	**Mean value £**	**SE**	**Gamma distribution Parameter α**	**Gamma distributionParameter β**	**Source**
**RS comparison**					
Prosthesis cost	2,808	N/A	N/A	N/A	NHS Supply Chain
Surgery costs (excluding prosthesis)	1,738	N/A	N/A	N/A	Vale *et al*.^25^
Hospital inpatient stay	1,628	N/A	N/A	N/A	Edlin *et al*.^26^
Follow-up cost post- RS	509	44	130	4	
Revision surgery	16,794	443	1435	12	Vanhegan *et al*.^4^
Post revision follow-up	400	30	169	2	Edlin *et al*.^26^
**THR comparison**							
Prosthesis cost (CeMoP)	1,575	N/A	N/A	N/A	NHS Supply Chain
Prosthesis cost (CeLCoC)	3,911	N/A	N/A	N/A	NHS Supply Chain
Prosthesis cost (CeCoP)	2,018	N/A	N/A	N/A	NHS Supply Chain
Surgery costs (excluding prosthesis)	1,738	N/A	N/A	N/A	Vale *et al*.^25^
Hospital inpatient stay	1,687	N/A	N/A	N/A	Edlin *et al*.^26^
Follow-up cost post- THR	400	30	169	2	
Revision surgery	16,794	443	1435	12	Vanhegan *et al*.^4^
Post revision follow-up	400	30	169	2	Edlin *et al*.^26^

*surgical mortality was the same for THR/RS and revision

The model was run probabilistically, with 1,000 iterations using standard distributions for input parameters in order to capture uncertainties [27]. Time horizons were 10 years and lifetime (to 100 years of age). Model outputs were mean quality-adjusted life years gained (QALYs), mean costs accumulated, the estimated probability that a particular prosthesis is cost-effective at a willingness to pay threshold of £20,000/QALY, and the incremental cost-effectiveness ratio (ICER in terms of cost/QALY) in scenarios where RS accumulated more QALYs at greater cost than THR.

### Health outcomes

Health outcomes were measured in QALYs [27]. Quality of life data were obtained from the patient reported outcome measures (PROMS) database for patients who had received a hip replacement between January 2009 and December 2012 [24]. EQ-5D scores for successful primary health and revision health states were adjusted for age and sex differences. The age-related utilities were assumed to be the same for the comparison of RS with THR (see previous publication for further information on PROMS analysis) [16]. In a sensitivity analysis we used the utility difference of RS versus THR reported by Edlin *et al*. (2012), and applied this for the first two years post-surgery [26]. (see S2 Table).

### Resource use and cost estimates

The NHS Supply Chain provided June 2013 costs for components of each prosthesis type and the total cost of each prosthesis (see previous publication for further information on THR prosthesis cost [16] and S3 Table for cost of RS prosthesis). We assumed the costs to be the same for the Birmingham Hip, Biomet and Finsbury prostheses due to lack of cost data for each individual prosthesis. The costs of a successful primary THR, RS, revision procedure and successful revision procedure were taken from the literature [4,25,26]. Costs of primary and revision THRs included surgery costs, prosthesis costs, hospital ward costs, and follow-up costs [4,25,26]. The cost of a successful revision procedure (£16,517) and cost of post-revision follow up (£394) were assumed to be the same for both RS and THR. The projected Health Services Cost Index (HSCI) was used to inflate costs to current prices (2014) [28].

### Sensitivity analyses

The base case utilized probabilistic sensitivity analysis to assess uncertainty in the model. Scenario analyses included the following: (a) changing the parametric distribution used to model lifetime revision rates (b) using device costs supplied by manufacturers to the National Institute for Health and Care Excellence for technology appraisal TA304 [29]; (c) using alternative quality of life utility values [26]. Additional scenarios investigated included the following: comparing the Birmingham Hip, Biomet and Finsbury male patient-device combinations that satisfy the NICE benchmark versus cemented metal on polythene device (CeMoP), cemented THR with ceramic on polythene bearing surface (CeCoP) and/or cementless ceramic on ceramic bearing surface (CeLCoC) THRs.

## Results

Six manufacturers of RS components accounted for almost all (96%) of RS interventions (S1 Fig). The Birmingham Hip contributed more than half of all RS use. Patient ASA grade and age distributions were similar between manufacturers (S2 Fig and S3 Fig). Usage of various head sizes was idiosyncratic and differed between the 6 manufacturers (S4 Fig).

**Fig 2:**
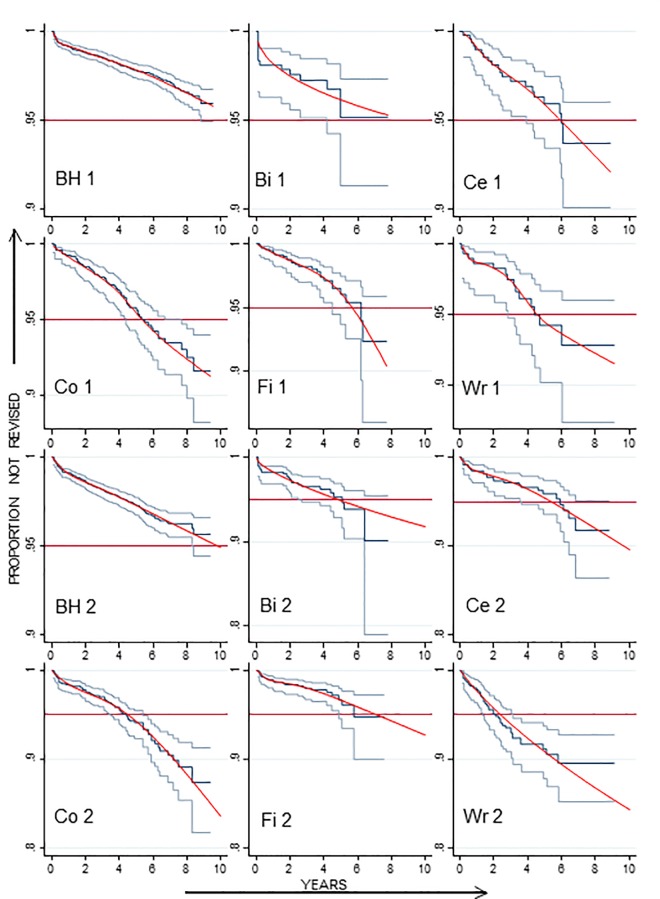
Kaplan-Meier revision plots (95% CI) and flexible parametric models for different manufacturer’s RS devices implanted in men graded as ASA 1 (upper panel) or ASA 2 (lower panel). BH = Birmingham Hip; Bi = Biomet; Ce = Centerpulse; Co = Corin; Fi = Finsbury; Wr = Wright UK.

Kaplan-Meier analyses indicated that for all six manufacturers, men experienced lower revision rates than women. However, even for men, the revision rate was poor for most manufacturers and flexible parametric models predicted a low probability of satisfying the 10 year NICE benchmark (Fig 2). The only device which delivered an overall revision rate within or near the 10 year benchmark was the Birmingham Hip. For women, no manufacturer’s device delivered a revision rate within the benchmark. All exceeded 5% revision within only 5 years, and by 10 years, predicted rates in women were all greater than 10% (S5 Fig).

In addition to RS device manufacturer and sex, the requirement for revision is likely to be influenced by the size of the head component and patient age [13]. We therefore estimated the predicted ten year revision rate for different head sizes using flexible parametric models with age as a covariate. Graphical examples are shown in S6–S9 Figs.

These flexible parametric models predicted poor 10 year performance for almost all female RS recipients irrespective of device head size, patient age at intervention or device manufacturer. Of the 6,646 women provided with RS, only about 2% were in head size-age-manufacturer categories likely to satisfy the NICE benchmark for revision. Of 18,720 male RS recipients, around 60% were estimated to be in such categories. Of these, 87% were Birmingham Hip recipients in head size categories of 50 mm or greater, and the rest were mostly recipients of Finsbury devices with head sizes ≤ 48mm or Biomet devices with a 50 to 54 mm head size.

**Fig 3:**
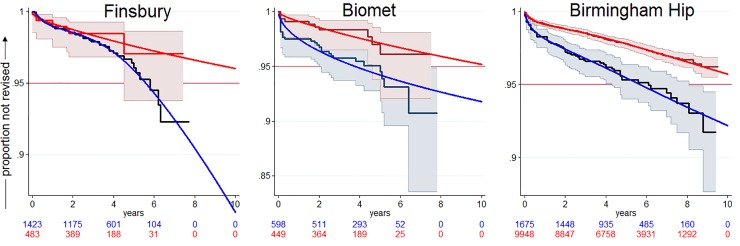
Estimated ten-year revision rates dichotomized by head size-patient combinations satisfying and not satisfying the ten year benchmark of 5%. The data are for male patients of ASA 1 plus ASA 2 grade receiving RS devices from different manufacturers (the subgroups within the NICE benchmark for revision were as follows: Finsbury (mean age 51.3 years): head sizes 48 or less and head size 50 if patient age was less than 50 years at intervention; Biomet (mean age 50.4 years): head size 50 if age less than 55 years, head sizes 52, and 54 if age less than 60 years; Birmingham Hip (mean age 55.5 years) head sizes 50 or greater and head size 48 if age less than 45 years). Note the more mature data for Birmingham Hip. For clarity the 95% CI of the Kaplan Meier plot for the Finsbury below-benchmark subgroup has been omitted.

Fig 3 shows revision performance for the Birmingham hip, Biomet and Finsbury RS devices in male ASA1 and ASA2 grade patients when head size-patient categories were dichotomized into those predicted to satisfy and not satisfy the NICE benchmark. Refinement by selecting for age within head sizes generally improved the revision profile.

The cost-effectiveness of the Birmingham Hip, Biomet and Finsbury devices satisfying the NICE benchmark for revision were compared with THR devices in male ASA 1 and ASA 2 grade patients. For the base case economic analysis we compared the most widely used RS (Birmingham hip, within NICE benchmark) with the most widely used THR device (CeMoP) in male grade ASA 1 and ASA grade 2 patients. Inputs for the economic analysis are summarised in Table 2. This analysis indicated that the probability that RS was cost-effective compared to THR remained low (<4%) at a willingness to pay of £20,000/QALY (for CEAC see S10 Fig). Birmingham Hip accumulated fewer lifetime QALYs for 40, 50 and 60 year old men; lifetime costs were greater than for RS by at least £2,900 and RS was dominated by THR for 60 year old men.

**Table 2:** Cost-effectiveness results for base case analysis Birmingham hip) versus CeMoP comparison for ASA1 plus ASA 2 male patients aged 40, 50 and 60 years.

	Age 40		Age 50		Age 60	
	BHIP	CeMoP	BHIP	CeMoP	BHIP	CeMoP
**Men: 10-year time horizon**						
Total mean costs £	12,211	10,348	12,133	10,033	12,024	9,829
Total mean QALYs	7.326	7.321	7.515	7.512	7.4850	7.4841
Incremental cost £	1,863		2,100		2,194	
Incremental QALYs	0.0054		0.0026		0.0009	
ICERs (£/QALY)	344,570		811,430		2,376,140	
**Men: Lifetime horizon**						
Total mean costs £	21,479	18,571	19,187	15,908	16,421	13,130
Total mean QALYs	16.587	16.581	14.706	14.705	12.109	12.110
Incremental cost £	2,908		3,278		3,291	
Incremental QALYs	0.0059		0.0013		-0.001	
ICERs (£/QALY)	488,836		2,493,847		Dominated	

Since the Birmingham Hip was the best performing RS device, we performed a sensitivity analysis in which it was compared with the best performing THR device (CeCoP). In this comparison the probability that RS was cost-effective was 46% for 40 year old men, and less for older men.

Since the mean age for the Birmingham Hip recipients was less than that for the CeMoP and CeCoP THR devices, we performed a further sensitivity analysis comparing the Birmingham Hip with the CeLCoC THR since this has been used in a younger population than the other THRs. In this comparison, the probability that the Birmingham Hip was cost-effective was ~76%. However, using alternative plausible models of lifetime revision resulted in a drop in probability of the Birmingham Hip being cost-effective to less than 10% due to poor predicted performance of RS.

We compared the Biomet RS and Finsbury devices (patient device combinations within the NICE benchmark for revision shown in Fig 3) with the best performing THR device (CeCoP) in male grade ASA 1 and ASA grade 2 patients, the probability that RS was cost-effective was <6% at a willingness to pay of £20,000/QALY.

When manufacturer’s device costs were used instead of NHS Supply Chain costs, the probability that the Birmingham Hip was cost-effective relative to the CeMoP THR device remained below 5%. In further sensitivity analyses, we applied the one year QOL advantage for RS reported by Edlin *et al*. (2012) in the Birmingham Hip versus CeMoP comparison; the Birmingham Hip was more costly and accumulated more QALYs, and the probability that Birmingham Hip was cost-effective was 100% for 40, 50 and 60 year old men (with NHS Supply Chain prosthesis costs) [26].

## Discussion

Conceptual advantages proposed for resurfacing relative to THR include preservation of bone and improved durability from metal-on-metal bearing surfaces; features leading to the perception that resurfacing is suitable for young and active patients. The age profile of RS recipients in National Joint Registry reflects this perception, but there is a lack of randomised or other comparative evidence to underpin this usage and, the observed revision rates for RS are alarmingly poor relative to THR. The single RCT so far conducted indicated a lack of short term advantage for RS in a mix of male and female patients with a mean age of 56 years [30]. National Joint Registry data for women who have received resurfacing devices highlights poor revision performance; even when the data are stratified according to device manufacturer, device head size and controlled for patient age, poor revision rates are still found and almost no subgroup of device-patient-head size combinations was predicted to satisfy the NICE benchmark for revision. This registry evidence points to a high probability of RS failure relative to THR, and suggests that resurfacing should be abandoned for women until good comparative evidence is available that shows that in young active women RS is at least as successful as THR.

For male recipients of RS devices it was possible to identify patient-head size-device manufacturer subgroups predicted to satisfy benchmark performance retrospectively. However, for five of the six manufacturers of the most commonly used devices, such subgroups represented less than half of device recipients. Only the Birmingham Hip, Biomet and Finsbury devices delivered appreciable proportions of patients predicted to perform within benchmark (85%, 43% and 25% respectively). When the cost-effectiveness of resurfacing relative to alternative THR prostheses was compared using these manufacturer device-patient subgroups, there appeared little likelihood that resurfacing in these groups has represented a cost-effective deployment of health provider resources. Furthermore, it is clear that without retrospective selection of better performing RS subgroups, the proposition that resurfacing should be continued for male patients on the grounds of cost-effectiveness cannot be supported.

### Strengths, limitations and previous analyses

The most significant limitation is the discrepancy in age distribution between resurfacing and THR recipients. This means that comparing the two types of intervention requires modelling THR revision rates outside of the observed age distribution, by controlling for age as a covariate. Unfortunately, there is no viable alternative to this approach because of the lack of comparative studies. A further difficulty is the inability to control for physical activity of patients or surgeon experience, which are likely to impact on the probability of device failure. Our approach to some of these difficulties has followed that of others in using flexible parametric models [5,12,13]. McMinn (2015) [31] has suggested that to gain experience in RS a surgeon would need to conduct about 1,000 operations; a large number in the context of the total interventions for some RS devices recorded in the NJR. Whether a similarly prolonged learning curve may be required for THR is unknown. McMinn has suggested poor performance of small cup size RS devices in female recipients may be attributable to their use by relatively inexperienced surgeons [31].

Several weaknesses in our cost-effectiveness analyses must be acknowledged, stemming from uncertainties about model input parameters. In particular, there is no information about long term quality of life following implantation of the different types of hip replacement of interest, or about long term device survival following the different implants examined here. The Alberta Hip Improvement Project study compared 3, 12 and 24 month SF-36 physical function changes from baseline for propensity matched Birmingham Hip and THR patients. In this study there was considerable missing data and a 3 month advantage for RS almost disappeared by 2 years [32]. A further limitation is that NHS Supply Chain costs are unlikely to perfectly reflect real world costs because of variable and individual contractual arrangements between NHS Trusts and prosthesis manufacturers, and potential economies of scale. Our analyses do not include costs for monitoring plasma cobalt levels; now recommended for patients following MOM implants, and this might slightly bias results in favor of RS [33–34]. Nevertheless, our analyses use IPD which reflect real world use of hip replacements in England and Wales over the recent decade, and provide estimates of lifetime cost-effectiveness that will be of interest to decision makers. Estimating life time revision rates requires modelling substantially beyond the observed data and the results are sensitive to the model chosen.

This is the first lifetime cost-effectiveness analysis using data from the National Joint Registry England and Wales on RS devices satisfying the ten year NICE benchmark. An early analysis by Vale *et al* (2002) compared RS with THR as a generalized intervention but was limited by the immaturity of available revision data [25]. Edlin *et al*. undertook cost-effectiveness analysis over a one year time horizon using cost and clinical data simultaneously collected from a randomized controlled trial [26]. This analysis did not capture important differences between devices in terms of long term revision requirements. Heintzbergen *et al*. (2013) undertook a 15 year analysis and compared the best performing RS device (Birmingham Hip) with the generality of THR devices, most of which were used for older patients [35]. This analysis tends to bias in favor of RS, and since RS is now mainly justified on the grounds of its potential long term benefit in delaying requirement for revision a longer time horizon appears more appropriate.

Like other cost-effectiveness analyses of hip replacement we assumed equal mortality for recipients of different devices [17, 36, 37,38]. Two recent UK observational studies proposed better survival after RS than THR, but the authors acknowledged their conclusions were problematic due to the influence of hidden confounders [10, 39].

## Conclusion

In conclusion, the cost-effectiveness case for the use of RS as a general class of intervention for young patients with osteoarthritis is weak. We would suggest that much of the past use of RS has probably been wasteful of NHS resources. Alternative THR devices are likely to be equally or more cost-effective. In the absence of good comparative evidence, RS is difficult to justify on the grounds of either cost-effectiveness or on the basis of preferable revision rates.

## Supporting Information

S1 Fig Relative past use of different RS devices according to RS head manufacturer. (DOCX)

S2 Fig ASA grade distribution of recipients of six major RS head manufacturers. (DOCX)

S3 Fig Age distribution of recipients of six major RS head manufacturers. (DOCX)

S4 Fig Device head size distribution for recipients of six major RS head manufacturers. (DOCX)

S5 Fig Kaplan-Meier revision plots (95% CI) with flexible parametric models for six major RS head manufacturer’s implanted in women graded as ASA 1 or ASA 2. (DOCX)

S6 Fig Female recipients (ASA grade 1 + 2) of the Birmingham hip of various head sizes. (DOCX)

S7 Fig Male recipients (ASA grade 1 + 2) of the Birmingham hip of various head sizes. (DOCX)

S8 Fig Male recipients (ASA grade 1 + 2) of the Biomet device of various head sizes. (DOCX)

S9 Fig Male recipients (ASA grade 1 + 2) of the Finsbury device of various head sizes. (DOCX)

S10 Fig Cost-effectiveness acceptability curves for Birmingham Hip versus CeMoP THR using bath tub fit ASA1 grade 40, 50, 60 year old men. (DOCX)

S11 Fig Cost-effectiveness scatter plots for Birmingham Hip versus CeMoP THR (using Bath tub fit) ASA1 grade 40, 50, 60 year old men. (DOCX)

S1 Table Characteristics of the five THR categories. (DOCX)

S2 Table Utility parameters for the sensitivity analysis using Edlin *et al*.(2012) utility difference of RS versus THR. (DOCX)

S3 Table Cost of RS prosthesis. (DOCX)

S4 Table Cost parameters for the sensitivity analysis using manufacturer’s device cost. (DOCX)
