# Supplementary material for: Has Metal-On-Metal Resurfacing Been a Cost-Effective Intervention for Health Care Providers?—A Registry Based Study
Source: PLoS One. 2016 Nov 1;11(11):e0165021. doi: 10.1371/journal.pone.0165021 (PMC5089767; doi:10.1371/journal.pone.0165021)
Supplement: S1 Fig — (DOCX) [file pone.0165021.s001.docx]

**S1 Figure.** Relative past use of different RS devices according to RS head manufacturer

Manufacturers: Biomet, Centerpulse, Comis Orthopaedics, Corin, DePuy, ESKA,Finsbury Implantcast GmbH, International orthopaedics, MATOrtho, Birmingham Hip, Soveriagn Medical, Stryker, Van Straten Medical, Wright Medical UK, Zimmer. Note: De Puy was excluded from subsequent analyses.
