# Supplementary material for: Has Metal-On-Metal Resurfacing Been a Cost-Effective Intervention for Health Care Providers?—A Registry Based Study
Source: PLoS One. 2016 Nov 1;11(11):e0165021. doi: 10.1371/journal.pone.0165021 (PMC5089767; doi:10.1371/journal.pone.0165021)
Supplement: S2 Fig — (DOCX) [file pone.0165021.s002.docx]

**S2 Figure.** ASA grade distribution of recipients of six major RS head manufacturers
