# Supplementary material for: Has Metal-On-Metal Resurfacing Been a Cost-Effective Intervention for Health Care Providers?—A Registry Based Study
Source: PLoS One. 2016 Nov 1;11(11):e0165021. doi: 10.1371/journal.pone.0165021 (PMC5089767; doi:10.1371/journal.pone.0165021)
Supplement: S5 Fig — (DOCX) [file pone.0165021.s005.docx]

**S5 Figure.** Kaplan-Meier revision plots (95% CI) with flexible parametric models for six major RS head manufacturer’s implanted in women graded as ASA 1 or ASA 2

ASA 1 (upper panel) & ASA 2 (lower panel)

BH= Birmingham Hip, Bi = Biomet, Ce = Centerpulse, Co = Corin, Fi = Finsbury, Wr = Wright UK
