# Supplementary material for: Has Metal-On-Metal Resurfacing Been a Cost-Effective Intervention for Health Care Providers?—A Registry Based Study
Source: PLoS One. 2016 Nov 1;11(11):e0165021. doi: 10.1371/journal.pone.0165021 (PMC5089767; doi:10.1371/journal.pone.0165021)
Supplement: S7 Fig — (DOCX) [file pone.0165021.s007.docx]

**S7 Figure.** Male recipients (ASA grade 1 + 2) of the Birmingham hip of various head sizes*

***** Kaplan Meier and flexible parametric models of revision for male recipients (ASA grade 1 + 2) of the Birmingham hip of various head sizes. For the parametric models age at primary intervention was covariate and revision was predicted for 40, 45, 50, 55, 60, 65 and 70 year old patients. The red horizontal line represents the NICE revision benchmark.
