# Supplementary material for: Has Metal-On-Metal Resurfacing Been a Cost-Effective Intervention for Health Care Providers?—A Registry Based Study
Source: PLoS One. 2016 Nov 1;11(11):e0165021. doi: 10.1371/journal.pone.0165021 (PMC5089767; doi:10.1371/journal.pone.0165021)
Supplement: S10 Fig — (DOCX) [file pone.0165021.s010.docx]

**S10 Figure.** Cost effectiveness acceptability curves for Birmingham Hip versus CeMoP THR (using bath tub fit) ASA1 grade 40, 50, 60 year old men
