# Supplementary material for: Has Metal-On-Metal Resurfacing Been a Cost-Effective Intervention for Health Care Providers?—A Registry Based Study
Source: PLoS One. 2016 Nov 1;11(11):e0165021. doi: 10.1371/journal.pone.0165021 (PMC5089767; doi:10.1371/journal.pone.0165021)
Supplement: S1 Table — (DOCX) [file pone.0165021.s012.docx]

S1 Table. Characteristics of the five THR categories

| **Type** | **Components** | **Acronym** | **Female patients** | **Male patients** |
| --- | --- | --- | --- | --- |
| **A** | **M**etal head (cemented stem) **o**n **Ce**mented **p**olyethylene cup | CeMoP | 83,813 | 41,472 |
| **B** | **M**etal head (cementLess stem) **o**n **Ce**ment**l**ess HA coated metal cup (**p**olyethylene liner) | CeLMoP | 22,819 | 15,055 |
| **C** | **C**eramic head (cementLess stem) **o**n **Ce**ment**l**ess HA coated metal cup (**c**eramic liner) | CeLCoC | 19,252 | 15,502 |
| **D** | **Hy**brid **M**etal head (cemented stem) **o**n cementless HA coated metal cup (**p**olyethylene liner) | HyMoP | 18,290 | 10,181 |
| **E** | **C**eramic head (cemented stem) **o**n **Ce**mented **p**olyethylene cup | CeCoP | 7,728 | 4,977 |

Ce= cemented CeL=cementless Hy=Hybrid P=polyethylene M=metal C=ceramic HA=hydroxyapetite

Please note that the numbers of patients refer to all ASA categories combined.
