# Supplementary material for: Has Metal-On-Metal Resurfacing Been a Cost-Effective Intervention for Health Care Providers?—A Registry Based Study
Source: PLoS One. 2016 Nov 1;11(11):e0165021. doi: 10.1371/journal.pone.0165021 (PMC5089767; doi:10.1371/journal.pone.0165021)
Supplement: S2 Table — (DOCX) [file pone.0165021.s013.docx]

**S2 Table.** Utility parameters used in a sensitivity analysis (Edlin *et al*.2012)

| Quality of life - 40 years male | RS | THR |
| --- | --- | --- |
| 1 – 2 years | 0.769 | 0.736 |
| Year 3+* | 0.736 | 0.736 |

* age-related utilities from PROMS
