# Supplementary material for: Has Metal-On-Metal Resurfacing Been a Cost-Effective Intervention for Health Care Providers?—A Registry Based Study
Source: PLoS One. 2016 Nov 1;11(11):e0165021. doi: 10.1371/journal.pone.0165021 (PMC5089767; doi:10.1371/journal.pone.0165021)
Supplement: S3 Table — (DOCX) [file pone.0165021.s014.docx]

S3 Table Cost of RS prosthesis (as reported by NHS supply chain)

| Component | Average unit cost (£) | Supplier list price (£) | | |
| --- | --- | --- | --- | --- |
|  |  | Supplier 1  (£) | Supplier 2 (£) | Supplier 3 (£) |
| Acetabular cup HA coated | 1,600 | 1,708 | 1,552 | 1,539 |
| Resurfacing head cemented | 1,042 | 1,152 | 874 | 1,101 |
| Mixing Bowl & cement | 165 | N/A | N/A | N/A |
| Total cost | 2,808 |  | | |
