# Supplementary material for: Has Metal-On-Metal Resurfacing Been a Cost-Effective Intervention for Health Care Providers?—A Registry Based Study
Source: PLoS One. 2016 Nov 1;11(11):e0165021. doi: 10.1371/journal.pone.0165021 (PMC5089767; doi:10.1371/journal.pone.0165021)
Supplement: S4 Table — (DOCX) [file pone.0165021.s015.docx]

S4 Table Cost parameters for the sensitivity analysis using manufacturer’s device cost

| **Category** | **Component** | **Average Unit cost (£)** |
| --- | --- | --- |
| C – CeLCoC | Cementless HAC stem | 503 |
|  | Ceramic head | 340 |
|  | Metal cup – cementless HA | 485 |
|  | Liner ceramic | 401 |
|  | **Total** | **1,731** |
| E – CeCoP | Cemented stem | 273 |
|  | Ceramic head | 340 |
|  | Polyethylene cup – cemented | 131 |
|  | Cemented stem & cup extras | 126 |
|  | **Total** | **872** |
| 1. CeMoP | Cemented stem | 273 |
|  | Metal head | 125 |
|  | Polyethylene cup – cemented | 131 |
|  | Cemented stem & cup extras | 144 |
|  | **Total** | **675** |
| RS | Resurfacing head components | 683 |
|  | Resurfacing Cup | 1,228 |
|  | Distal centraliser | 36.00 |
|  | Femoral pressuriser | 18.00 |
|  | Cement cup extras | 91 |
|  | **Total** | **£2,056** |
